# *Salmonella* Typhi Bactericidal Antibodies Reduce Disease Severity but Do Not Protect against Typhoid Fever in a Controlled Human Infection Model

**DOI:** 10.3389/fimmu.2017.01916

**Published:** 2018-01-17

**Authors:** Helene B. Juel, Helena B. Thomaides-Brears, Thomas C. Darton, Claire Jones, Elizabeth Jones, Sonu Shrestha, Rebecca Sie, Andrew Eustace, Ushma Galal, Prathiba Kurupati, Tan T. Van, Nga T. V. Thieu, Stephen Baker, Christoph J. Blohmke, Andrew J. Pollard

**Affiliations:** ^1^Oxford Vaccine Group, Department of Paediatrics, University of Oxford, The NIHR Oxford Biomedical Research Centre, Oxford, United Kingdom; ^2^Statens Serum Institut, Copenhagen, Denmark; ^3^Department of Infection, Immunity and Cardiovascular Disease, University of Sheffield, Sheffield, United Kingdom; ^4^Nuffield Department of Primary Care Health Sciences, Clinical Trials Unit, University of Oxford, Oxford, United Kingdom; ^5^Weatherall Institute of Molecular Medicine, University of Oxford, Oxford, United Kingdom; ^6^The Hospital for Tropical Diseases, Wellcome Trust Major Overseas Programme, Oxford University Clinical Research Unit, Ho Chi Minh City, Vietnam; ^7^Centre for Tropical Medicine and Global Health, Nuffield Department of Medicine, University of Oxford, Oxford, United Kingdom; ^8^The Department of Medicine, University of Cambridge, Cambridge, United Kingdom

**Keywords:** typhoid infection, bactericidal activity, human challenge model, *Salmonella enterica* Typhi, immune responses

## Abstract

Effective vaccines against *Salmonella Typhi*, a major cause of febrile illness in tropical regions, can have a significant effect as a disease control measure. Earlier work has shown that immunization with either of two *Salmonella* Typhi vaccines, licensed Ty21a or candidate M01ZH09, did not provide full immunity in a controlled human infection model. Here, we describe the human humoral immune responses to these oral vaccines and their functional role in protection after challenge with *S*. Typhi. Serum, obtained from healthy volunteers before and after vaccination with Ty21a or M01ZH09 or placebo and before and after oral challenge with wild-type *S*. Typhi, was assessed for bactericidal activity. Single-dose vaccination with M01ZH09 induced an increase in serum bactericidal antibodies (*p* = 0.001) while three doses of Ty21a did not. No association between bactericidal activity and protection against typhoid after challenge was seen in either vaccine arm. Bactericidal activity after vaccination correlated significantly with delayed disease onset (*p* = 0.013), lower bacterial burden (*p* = 0.006), and decreased disease severity scores (*p* = 0.021). Depletion of antibodies directed against lipopolysaccharide significantly reduced bactericidal activity (*p* = 0.009). We conclude that antibodies induced after ingestion of oral live-attenuated typhoid vaccines or after challenge with wild-type *S*. Typhi exhibit bactericidal activity. This bactericidal activity is mediated by anti-O:LPS antibodies and significantly reduces clinical symptoms but does not provide sterile immunity. This directs future vaccine studies toward other antigens or mechanisms of protection against typhoid.

## Introduction

Typhoid fever is a systemic infection caused by *Salmonella enterica* serovar Typhi (*S*. Typhi) and results in significant morbidity, with an estimated 11.9–26.9 million infections occurring each year (1, 2). The use of vaccination as a public health prevention measure may alleviate significant disease burden, especially as infection arises *via* human-to-human transmission and there is no known environmental reservoir (3). Currently, both subunit and live oral vaccines against *S*. Typhi are licensed and recommended for use in high-incidence settings by the WHO (4). The protective efficacy of the Vi (virulence) capsular polysaccharide vaccine in the first year following vaccination is ~69% (5, 6), whereas the live oral Ty21a vaccine offers ~35% protection (5, 7). Neither vaccine is licensed for use in young children and, as one-third of the cases occur under the age of five (1), the development of new vaccines is vital.

To enable the development and evaluation of next-generation typhoid vaccines, improved characterization of the nature of protection afforded by vaccination is required. While some protection against typhoid is thought to be conferred by cell-mediated immunity (8), evidence from serosurveillance and vaccine efficacy trials indicates that O lipopolysaccharide (O:LPS) and flagellin (H) antigens are immunogenic in a dose-dependent manner, and that anti-Vi IgG antibody is protective (6, 9). Further description of the functional role of these antibodies during infection has been evaluated in murine models of *S*. Typhimurium infection (10) and through *in vitro* studies after immunization (11, 12). While these studies suggest that antibodies against both Vi and O9:LPS have direct bactericidal activity in the presence of complement, or opsonize ahead of pathogen phagocytosis, extrapolation of such data to a human infection with *S*. Typhi has limitations (13).

Using a controlled human infection model (CHIM) of typhoid fever, the protective efficacy of two live oral attenuated typhoid vaccines (Ty21a and M01ZH09) was recently assessed relative to placebo (14, 15). Ty21a, a licensed vaccine, and M01ZH09, a candidate vaccine, are derived from the parent strain Ty2. M01ZH09 was constructed by defined, independently attenuated deletion of the *ssaV* and *aroC* genes and expresses Vi at negligible levels based on immunogenicity measurements (14, 16). Ty21a contains additional genetic attenuations and does not constitutively express Vi (17). Neither a single-dose M01ZH09 immunization nor three doses of Ty21a provided full immunity in a model where diagnosis of typhoid disease (TD) was defined as confirmed *S*. Typhi bacteremia or by ≥12 h of sustained fever ≥38°C (14, 15). Here, we explored the functionality of antibodies induced by oral vaccination and the association with protection against typhoid in the challenge model.

## Materials and Methods

### Ethics and Approvals

A randomized, double-blind, placebo-controlled trial was performed at the Centre for Clinical Vaccinology and Tropical Medicine, Churchill Hospital, Oxford, UK (clinicaltrials.gov NCT679172; EudraCT 2011-000381-35) (14). This study was carried out in accordance with the recommendations of the Oxford University Clinical Trials and Research Governance Department, and the protocol approved by NRES South Central––Oxford A (11/SC/0302) and conducted in accordance with the declaration of Helsinki (2008) and the International Conference of Harmonization of Good Clinical Practice guidelines. An independent Data Monitoring and Safety Committee monitored the trial. To perform the assays described, human complement was collected in a parallel, prospective observational study (clinicaltrials.gov NCT01945307; 13/SC/0375). All subjects gave written informed consent in accordance with the Declaration of Helsinki.

### Study Design, Vaccination, and Challenge

The study design and results of the primary study objectives of the clinical trial have been reported previously (14). Briefly, 99 adult participants were randomly assigned in the ratio 1:1:1 to three study arms: (1) vaccine (a single oral dose of M01ZH09 vaccine containing 1 × 10^10^ CFU live attenuated *S*. Typhi M01ZH09 strain), (2) placebo, and (3) comparator (three doses of Ty21a capsules each containing 2 × 10^9^ CFU). Participants were challenged with 10^4^ CFU of wild-type *S*. Typhi Quailes strain 4 weeks after vaccination (D0) (15). Antibiotic treatment was initiated at typhoid diagnosis (TD) or 14 days after challenge in those participants who were not diagnosed with typhoid fever (nTD). Diagnosis of TD was defined as confirmed *S*. Typhi bacteremia or by ≥12 h of sustained fever ≥38°C (14, 15) and full protection was defined as not meeting the criteria for diagnosis (nTD).

### Serum Bactericidal Antibody Assay

To prepare a source of human complement, fresh serum from a UK healthy adult donor was depleted of *S*. Typhi-binding antibodies that may otherwise influence the performance of complement in our assays, as modified from Hart *et al*. (11, 18). Following three rounds of adsorption with 10^10^ CFU of log phase *S*. Typhi Quailes per 1 mL of serum, significant depletion of anti-Vi and anti-O:LPS antibody titers was achieved and demonstrated by ELISA (Table S1 in Supplementary Material), without change in complement activity, as measured by CH100 and AP100 hemolytic assays (data not shown). To prepare aliquots of log-phase *S*. Typhi Quailes for the serum bactericidal antibody (SBA) assay, an overnight culture, set up from a single colony in Luria Bertani broth (LB), was diluted 33-fold in LB supplemented with 10% sucrose. This was grown at 37°C shaking to achieve 3.5 × 10^8^–3.5 × 10^9^ CFU/mL and frozen in aliquots ahead of use in SBA.

Serum samples were evaluated in the SBA from a subset of participants from whom serum was available and representing similar numbers across all study arms over multiple time points (Placebo *n* = 18, Ty21a *n* = 16, M01ZH09 *n* = 18, TD *n* = 30, nTD *n* = 22). Time points were at pre-vaccination (D28), at challenge (D0), and post-challenge days 28 (D28), 60 (D60), and 180 (D180). Briefly, serum samples were de-complemented by heat-inactivation and diluted before addition of 200 CFU of log phase *S*. Typhi Quailes in Hanks Buffered Saline Solution (HBSS; Life Technologies Ltd., UK) supplemented with 0.5% Bovine Serum Albumin (BSA; Sigma-Aldrich, UK). *S*. Typhi-specific antibody-depleted human complement serum was added to a final complement concentration of 25% and bacteria incubated for 1 h at 37°C with shaking before plating on tryptic soya agar (TSA) plates (Oxoid Ltd., UK). In each experiment, complement serum and heat-inactivated complement serum alone were included as controls for bacterial survival; test samples without complement were included to assess any complement-independent killing; and a positive control, from another UK healthy donor, to assess inter-plate variability. Only experimental plates in which the positive control was within twofold of its defined SBA titer and with >60% overlap in CFU counts between the complement only and heat-inactivated complement only controls were accepted. The SBA titer was defined as the reciprocal lowest dilution of test serum to achieve ≤50% bacterial killing relative to complement alone. The slide agglutination test using *Salmonella* Vi anti-sera (Oxoid Ltd., UK) confirmed that Vi was expressed in the bacterial working stock. In nTD participants bactericidal activity a month after challenge (D28) was not measured, as preliminary experiments confirmed complete bactericidal activity consistent with residual ciprofloxacin in the samples as expected based on its pharmacokinetic profile (D28 coincided with the last day of antibiotic treatment for this subset) (19).

### Antigen Sources and Purification

Lipopolysaccharide (O9:LPS) was from Sigma-Aldrich, UK (L2387, >99% free of protein) and Vi capsular polysaccharide was from Sanofi Pasteur, UK (Typhim Vi^®^). Flagellin (H) was purified from a flagellin-deficient *S*. Enteritidis strain that expresses *S*. Typhi H:d from a plasmid (University of Maryland, USA) (14, 20). Cytolethal distending toxin B (CdtB) and pilus control protein L (PilL) coding sequences lacking transmembrane domains were PCR-amplified from *S*. Typhi CT18 strain genomic DNA, cloned into plasmid pET28b(+) (Novagen, UK) and expressed in *Escherichia coli* BL21(DE3)pLysS (Promega, WI, USA). Recombinant His-tagged CdtB and PilL were purified by tagging with nickel-coated agarose beads (Ni-NTA, Invitrogen) and elution from gravity flow columns (Qiagen, Germany). CdtB was renatured in 50-mM sodium phosphate solution and 500-mM NaCl. *hlyE* was PCR-amplified from Ty21a, cloned into pET21a vector (Novagen, UK), and expressed in an LPS-modified *E. coli* BL21 strain to produce endotoxin free proteins (Lucigen, ClearCoil BL21 cells). Recombinant His-tagged hemolysin E (HlyE) was purified using HisTrap nickel-affinity column (GE Healthcare) followed by desalting on a HiPrep 26/10 (GE Healthcare).

### ELISA

Immunoglobulin G (IgG), IgA, and IgM isotype responses to O9:LPS, H, HlyE, CdtB, and PilL were measured in serum as previously described (15, 21), with some modifications regarding antigen source and controls. Nominal ELISA units were calculated based on standard curves derived from pooled sera collected from the highest responders to O-antigen following vaccination with M01ZH09 (Emergent BioSolutions, Reading, UK). In addition, Vi-specific IgG were measured at D28, D0, and D28 using a commercial ELISA kit (VaccZyme™ Human anti-*S*. Typhi Vi IgG Enzyme Immunoassay Kit, The Binding Site Ltd., Birmingham, UK) following the manufacturer’s instructions.

### Antigen-Specific and Isotype-Specific Antibody-Depletion of Sera

In a subset of samples representing all study arms and both TD and nTD groups, antibodies specific to H or O9:LPS, or total IgA or IgG antibodies were depleted from the serum to assess the contributions of each of these antibodies to the bactericidal activity in sera. Antibodies specific to H or O9:LPS were adsorbed on to 96-well Maxisorp plates (Nunc, Copenhagen, Denmark) pre-coated with 1 µg/mL H or 15 µg/mL O9:LPS antigen in carbonate bicarbonate buffer (Sigma, UK), for eight rounds of 30-min incubations. A plate coated with BSA was used to create mock depleted controls. Total IgA and IgG antibodies were depleted using the Dynabeads Antibody coupling kit (Life Technologies Limited, UK). Briefly, each diluted serum sample was incubated for 1 h with 2-mg magnetic beads coated with either 10 µg of goat polyclonal IgG antibody raised against human IgA or IgG (AbD Serotec, UK), or an goat polyclonal IgG (Santa Cruz Biotechnology, USA). The depletions resulted in approximately 56% (H), 87% (O9:LPS), 72% (IgA), and 93% (IgG) median reduction in the antibody titer, as measured with ELISA.

### ELISpot

Antibody-secreting cell (ASC) responses to LPS, H, and Vi were measured at D28, 7 days after vaccination (D21), before challenge (D0), Day 7 after challenge (D7), and 48 h after the typhoid diagnosis visit (TD + 48) by ELISpot assay, as described in Ref. (14). Spots were photographed using the AID ELISpot optical reader (AID ELR03m, Autoimmun Diagnostika GmbH, Germany), manually counted by two observers, and expressed as spots per 10^6^ PBMCs. Zero ASC counts were given a nominal value of 0.1 and 0.1 added to all other observations.

### Clinical and Microbiological Data Collection

Daily clinical review and temperature measurements were carried out for 7 days after vaccination and 28 days after challenge. In addition, participants collected symptom data daily (solicited for headache, feeling generally unwell, loss of appetite, abdominal pain, nausea/vomiting, myalgia, arthralgia, cough, diarrhea and constipation, and any unsolicited symptoms) (14). Symptom severity scores were calculated by summing maximum severity values assigned to each of up to 10 symptoms experienced daily during 14 days after challenge and deriving the mean (14).

Blood (10 mL) samples were collected for bacterial culture at each visit. Cultures were performed by the local hospital accredited pathology laboratories according to national standard operating procedures (22–24), and as previously described (14, 15). Quantitative blood culture samples were collected immediately prior to antibiotic treatment by inoculation of 10-mL blood into an ISOLATOR 10 tube (Alere, UK).

### Cytokine Quantification

Cytokines were quantified in lithium-heparin plasma using a multiplex bead-based ELISA (Milliplex^®^ Human cytokine/chemokine magnetic bead panel, Merck Millipore, UK), following the manufacturer’s protocol with dilution of beads by twofold. Analytes tested were as follows: EGF, Fractalkine (CX3CL1), GROα (CXCL1), IFNα2, IFNγ, IL1β, IL1RA, IL2, IL6, IL8 (CXCL8), IL10, IL12p40, IL15, IL17A, IP10 (CXCL10), sCD40L, TGFα, TNFα, and VEGF. All samples were run in duplicate and were measured on a Luminex MAGPIX instrument (Luminex, Netherlands). Values were calculated from the average of duplicates which were above cytokine sensitivity limits and whose %CV was <30%.

### Statistical Analysis

SBA titers, ELISA antibody levels, and ASC counts were log_10_-transformed before statistical analysis. Within groups, the log_10_-transformed data were still skewed so non-parametric tests were used. All reported *p*-values are based on two-sided tests, with significance assumed at *p* < 0.05, without multiple testing corrections. Clinical, laboratory, and immunological data were collated using a clinical-trial database (OpenClinica, version 3.1). Data analysis was performed using R version 3.2.1 (25).

## Results

### Kinetics of Bactericidal Antibody Induction and Decay

**Figure 1:**
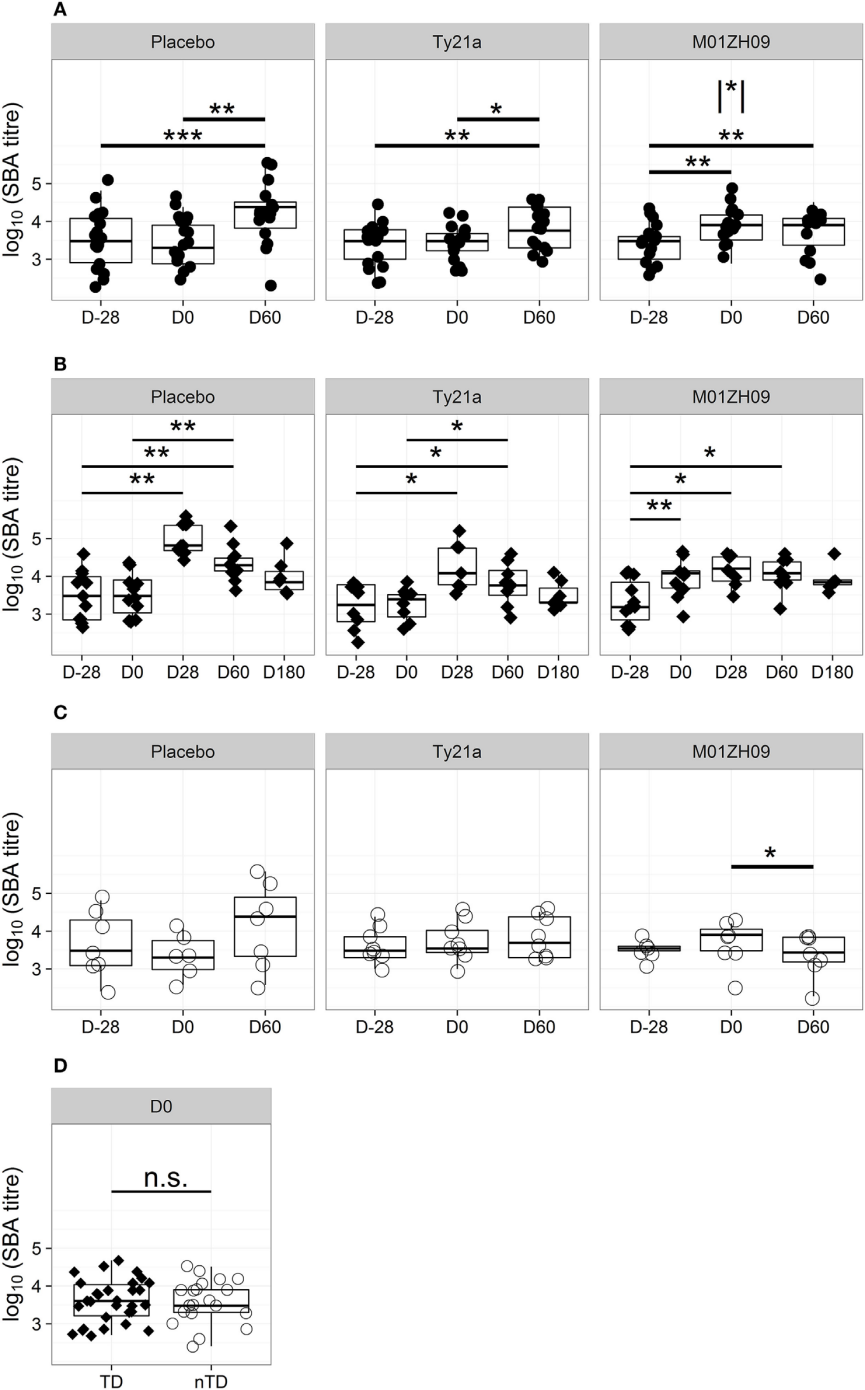
Kinetics of bactericidal antibody induction and decay. **(A)** Serum bactericidal antibody (SBA) titers in each study arm at D28 (pre-vaccination baseline), D0 (day of challenge), and D60 after challenge. **(B)** SBA titer kinetics after challenge in TD participants (filled diamonds) from each study arm. **(C)** SBA titers on D28, D0, and D60 after challenge in each study arm, shown for those with no typhoid diagnosis (nTD, empty circles). **(D)** SBA titers on the day of challenge, in those with typhoid diagnosis (TD) and nTD across all study arms. n.s., not significant, * *p* < 0.05; ** *p* < 0.01; *** *p* < 0.001. Asterisk within vertical lines indicates significance of pair-wise comparison between M01ZH09 and Ty21a vaccine arms for D0. Significance was determined by Wilcoxon signed rank test for paired value comparisons and by Mann–Whitney *U*-tests between groups.

Sera collected pre-vaccination in all study participants showed bactericidal activity against wild-type *S*. Typhi, with a 2–log_10_ range in titers but without significant differences between vaccine arms (Figure 1A; Table S2 in Supplementary Material). One month after vaccination, at D0, no increase in bactericidal activity was observed in either placebo or Ty21a recipients (Figure 1A). In contrast, a significant increase in bactericidal activity was measured 28 days after M01ZH09 vaccination (*p* = 0.001). This increase was principally attributable to the subset of M01ZH09 recipients who subsequently went on to be TD after challenge (*p* = 0.009, Figure 1B).

To identify differences in the immune responses induced by attenuated vaccines and wild-type *S*. Typhi, the effect of exposure to the challenge agent on levels of bactericidal antibody activity was examined. Sixty days after challenge bactericidal antibody activity was significantly higher than on D0 in the placebo and Ty21a arms in participants with TD after challenge (*p* = 0.009 and *p* = 0.035, respectively; Figure 1B). The bactericidal activity at D60 in sera from nTD placebo and nTD Ty21a recipients showed marked variability in inter-individual responses. In contrast, D60 titers in M01ZH09 recipients remained unchanged from D0 in TD participants and waned in nTD participants (*p* = 0.036; Figure 1C).

Evaluation of bactericidal activity in sera from TD individuals over a longer time course confirmed that exposure to the challenge strain-induced bactericidal antibodies only in placebo and Ty21a recipients (Figure 1B). In both groups, titers peaked at D28 after challenge and returned to near pre-vaccination baseline levels by D180. M01ZH09-vaccinated TD participants had bactericidal titers persistently higher than baseline for at least 60 days after challenge, despite the apparent absence of any boosting effect by exposure to/infection with wild-type challenge.

### The Impact of Bactericidal Antibody Activity on Challenge Outcome

We hypothesized that the presence of circulating bactericidal antibodies on the day of challenge may have a protective effect on challenge outcome. However, comparison of the bactericidal antibody titers between TD and nTD participants at D0 demonstrated no significant difference in protection (Figure 1D), even when assessed by vaccine allocation (Figure S1A in Supplementary Material). The similar titer ranges between TD and nTD groups indicated that serum bactericidal activity at D0, resulting either from preexisting exposure or vaccine induction, did not protect against TD.

**Figure 2:**
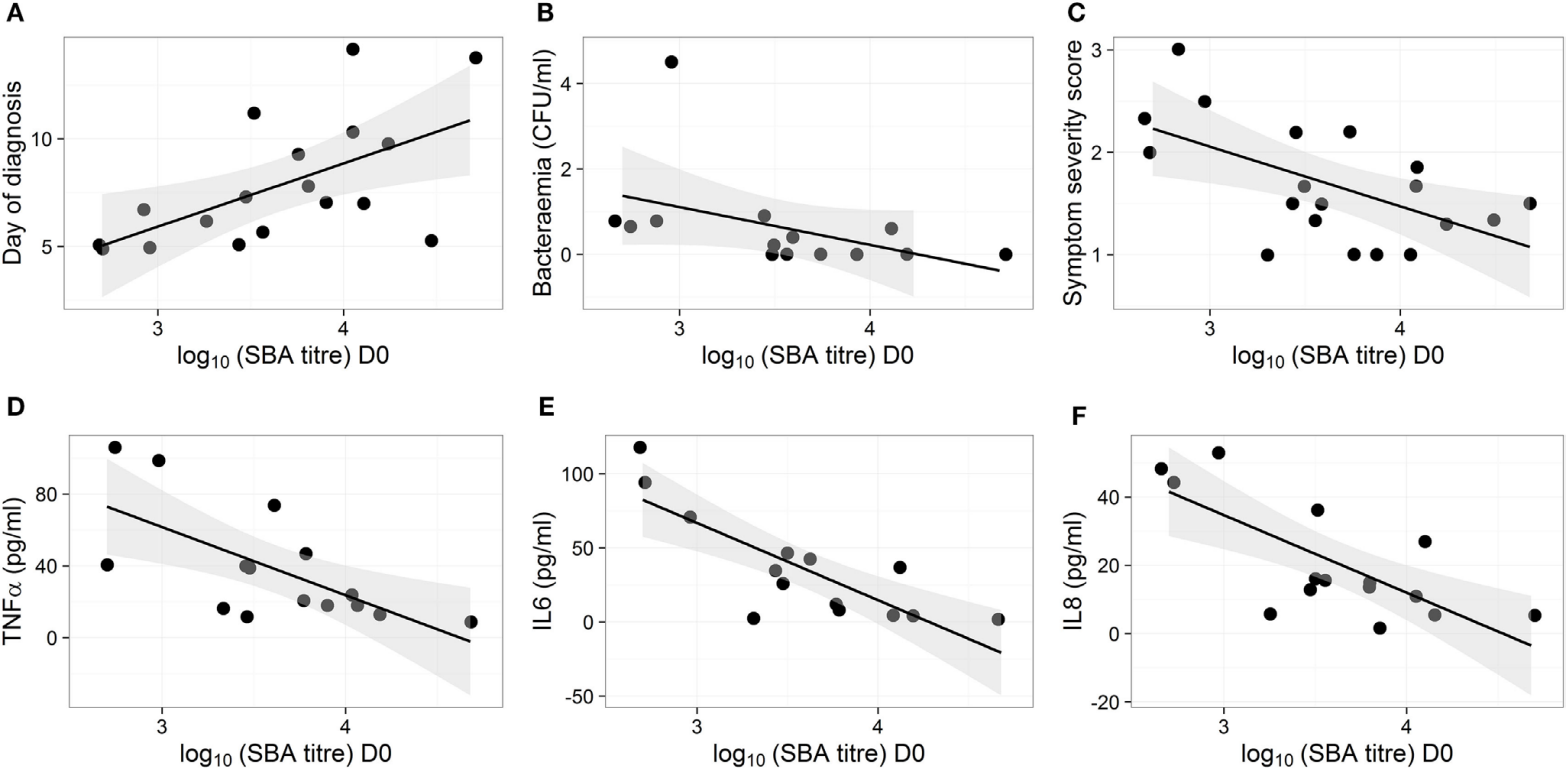
Bactericidal antibodies do not protect against typhoid infection but high bactericidal antibody activity reduces disease severity and cytokine response in vaccinated participants. **(A–F)** Correlation of disease parameters and plasma cytokine levels after diagnosis with SBA titers on D0 in participants from the Ty21a and M01ZH09 vaccine arms. Shown are day of diagnosis **(A)**, bacterial load in blood on the day of diagnosis **(B)**, symptom severity score **(C)**, and maximum concentrations of cytokines TNFα, IL6, and IL8 [**(D–F)**, respectively], in plasma samples collected within 48 h from diagnosis.

The D0 bactericidal antibody titers in TD participants were positively correlated, however, with time to typhoid diagnosis after challenge, when vaccination arms were combined (Figure 2A). Furthermore, D0 bactericidal antibody titer in these participants negatively correlated with quantitative bacterial blood count at diagnosis and with cumulative symptom severity score following challenge, but not with temperature (Figures 2B,C; Table 1). We found no significant correlations when placebo TD was similarly analyzed (Table 1). Taken together, these observations suggest that higher bactericidal activity delayed the onset of infection and reduced disease severity in vaccinated participants.

**Table 1:** Statistical output following correlations of D0 bactericidal titers with clinical and immunological data in TD groups.

	Correlation statistics: [*n*] Spearman rho (*p*-value)				
Parameter	All study arms	M01ZH09 + Ty21a	M01ZH09	Ty21a	Placebo
Day of diagnosis^a^	[29] 0.28 (0.145)	**[18] 0.58 (0.013)**	[11] 0.23 (0.502)	[7] 0.73 (0.062)	[11] −0.25 (0.466)
Bacterial count^b^	[23] −0.20 (0.362)	[14] −**0.69 (0.006)**	[7] −0.67 (0.102)	[7] −0.59 (0.168)	[9] 0.10 (0.796)
Symptom severity^c^	**[30]** −**0.48 (0.007)**	[19] −**0.52 (0.021)**	[11] −0.53 (0.095)	[8] −**0.73 (0.041)**	[11] −0.11 (0.746)
Max temperature^d^	[30] −0.25 (0.178)	[19] −0.26 (0.277)	[11] −0.09 (0.792)	[8] −**0.74 (0.038)**	[11] −0.15 (0.649)
Max temperature increase^e^	[30] −0.19 (0.314)	[19] −0.18 (0.453)	[11] −0.03 (0.925)	[8] −0.47 (0.24)	[11] −0.19 (0.569)
TNF-α^f^	**[20]** −**0.58 (0.007)**	[15] −**0.60 (0.019)**	**[7]** −**0.86 (0.014)**	[8] −0.48 (0.227)	[5] −0.56 (0.322)
IL-6^f^	[19] −0.20 (0.362)	[14] −**0.71 (0.005)**	**[7]** −**0.84 (0.019)**	[7] −0.66 (0.111)	[5] 0.21 (0.740)
IL-8^f^	**[20]** −**0.48 (0.007)**	[15] −**0.69 (0.004)**	**[7]** −**0.88 (0.019)**	[8] −**0.74 (0.038)**	[5] −0.05 (0.935)
IL-1RA^f^	[20] −0.30 (0.191)	[15] −0.51 (0.051)	[7] −0.73 (0.064)	[8] −0.18 (0.668)	[5] 0.05 (0.934)
IFN-γ^f^	[20] −0.17 (0.467)	[15] −0.45 (0.091)	**[7]** −**0.76 (0.046)**	[8] −**0.77 (0.025)**	[5] −0.41 (0.493)
CXCL10^f^	[20] −0.12 (0.618)	[15] −0.30 (0.271)	[7] −0.47 (0.284)	[8] −0.58 (0.133)	[5] 0.41 (0.493)
Max CRP^g^	[30] 0.06 (0.758)	[19] −0.09 (0.714)	[11] −0.28 (0.403)	[8] −0.52 (0.188)	[11] −0.01 (0.968)

*Correlations with statistical significance are shown in bold*.

*^a^Day of diagnosis based on S. Typhi confirmed bacteremia or by ≥12 h of sustained fever of ≥38°C*.

*^b^CFU/mL per bacterial quantification of 10-mL blood sample collected on day of diagnosis*.

^c^Symptom severity score, a cumulative score of solicited symptoms as defined in the Section “Materials and Methods.”

*^d^Maximum temperature recorded during the challenge period*.

*^e^Maximum temperature increase during the challenge period*.

*^f^Maximum cytokine concentration in plasma samples collected within 48 h of diagnosis*.

*^g^Maximum C-reactive protein concentration recorded during the challenge period*.

The concentrations of various plasma cytokines associated with the acute phase of infection after *S*. Typhi challenge (26) were measured and analyzed to evaluate the impact of bactericidal activity on other events in disease pathogenesis. TNFα, IL6 and IL8 were negatively correlated with the D0 bactericidal titers in the vaccinated participants (Figures 2D–F; Table 1). However, CRP did not correlate with D0 titers (Table 1). There was no association between D0 titers and these cytokines for the placebo group (Table 1). These data corroborate an effect of bactericidal antibody activity in reducing the inflammatory response during infection in live oral vaccine recipients. This was further supported by a vaccine subgroup analysis, which demonstrated stronger correlations in the M01ZH09 arm in whom the highest D0 bactericidal titers were observed (Table 1).

### Anti-LPS Antibodies Are the Key Mediators of Bactericidal Activity

The concentrations of IgG and IgA to assorted *S*. Typhi antigens were measured to elucidate the antigen specificity of functional antibodies. Other than O9:LPS, flagellin, and Vi polysaccharide that are known key *S*. Typhi surface epitopes, we also tested responses to hemolysin E (HlyE), cytolethal distending protein subunit B (CdtB) and pilus control protein L (PilL). In antigen array experiments these antigens were previously found to be the dominant targets of circulating antibodies in patients with acute typhoid fever (27, 28). Negligible anti-Vi titers were observed in almost all tested samples (Figure S2A in Supplementary Material). In a mixed-effects model, we identified O9:LPS as being the most likely antigen targeted by bactericidal antibody with some activity also shown to flagellin (Table 2).

**Table 2:** Estimates and *p*-values from linear mixed-effects models of SBA data from all arms and time points to ELISA titers against specific antigens, taking into account random effects for grouping multiple time points from the same participants.

Parameters	Estimate (95% CI)	*p*-Value
O lipopolysaccharide (O9:LPS) IgG	0.5957 (0.3, 0.9)	7.30E−05
O lipopolysaccharide (O9:LPS) IgA	0.9531 (0.7, 1.2)	1.60E−09
O lipopolysaccharide (O9:LPS) IgM	0.8876 (0.7, 1.1)	1.10E−13
Flagellin (H) IgG	0.3703 (−0.1,0.8)	0.123
Flagellin (H) IgA	0.5031 (0.1,1.0)	0.036
Flagellin (H) IgM	0.3078 (0.2, 0.4)	1.40E−06
Vi IgG	0.2538 (−0.1, 0.6)	0.109
HlyE IgG	0.6144 (0.1, 1.2)	0.037
HlyE IgA	0.3073 (−0.2, 0.8)	0.223
CdtB IgG	0.0978 (−0.4, 0.6)	0.718
CdtB IgA	−0.0678 (−0.8, 0.6)	0.855
PilL IgG	−0.0433 (−0.5, 0.4)	0.843
PilL IgA	−0.1738 (−0.8, 0.5)	0.619

**Figure 3:**
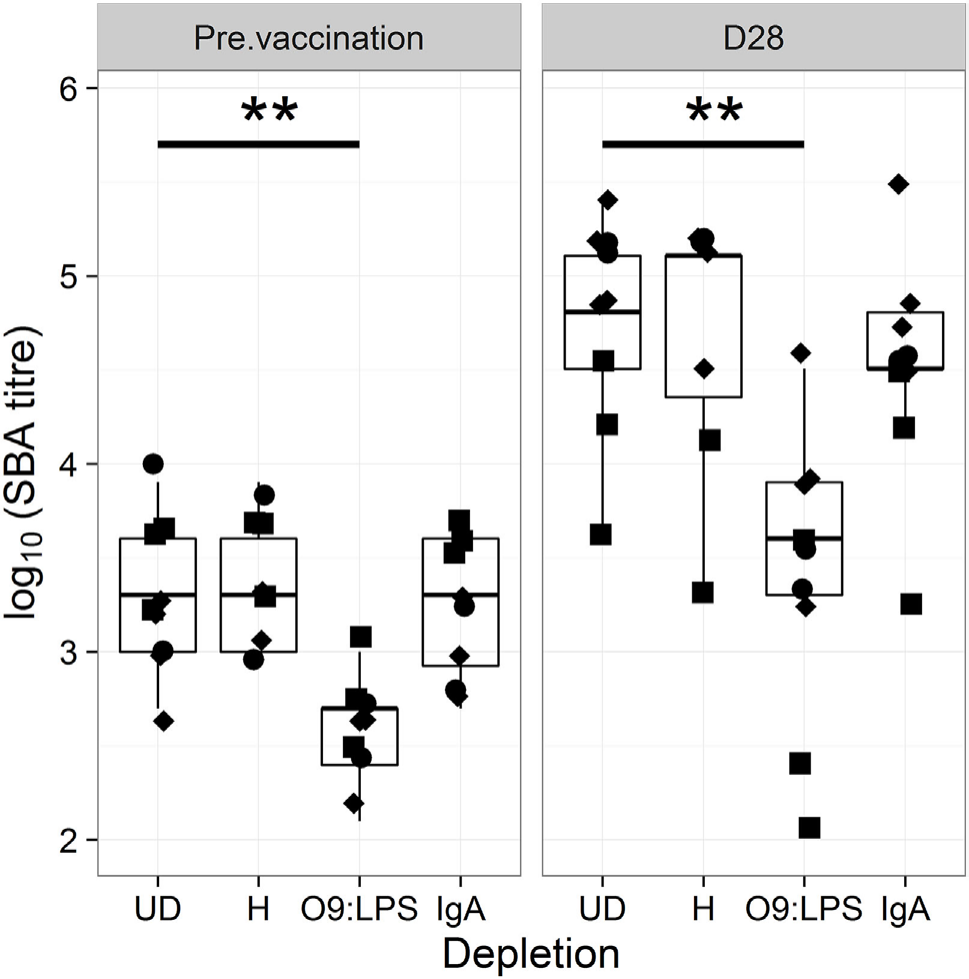
Bactericidal antibodies are specific to O9:LPS. SBA titer pre-vaccination (D28) and at D28 after challenge of samples undepleted (UD), or depleted of anti-H, anti-O9:LPS or total IgA antibodies. Diamonds indicate placebo, circles indicate Ty21a and squares indicate M01ZH09 samples. Significance was determined by Wilcoxon signed rank tests.

For confirmation, we depleted sera of antigen-specific antibodies prior to assessing bactericidal activity. As shown in Figure 3, depletion of O9:LPS antibodies resulted in significant reduction in bactericidal titers from samples at baseline or at D28 (both *p* = 0.009), independent of subset. In contrast, depleting antibodies specific to flagellin, or total IgA antibodies, did not affect activity (Figure 3).

**Figure 4:**
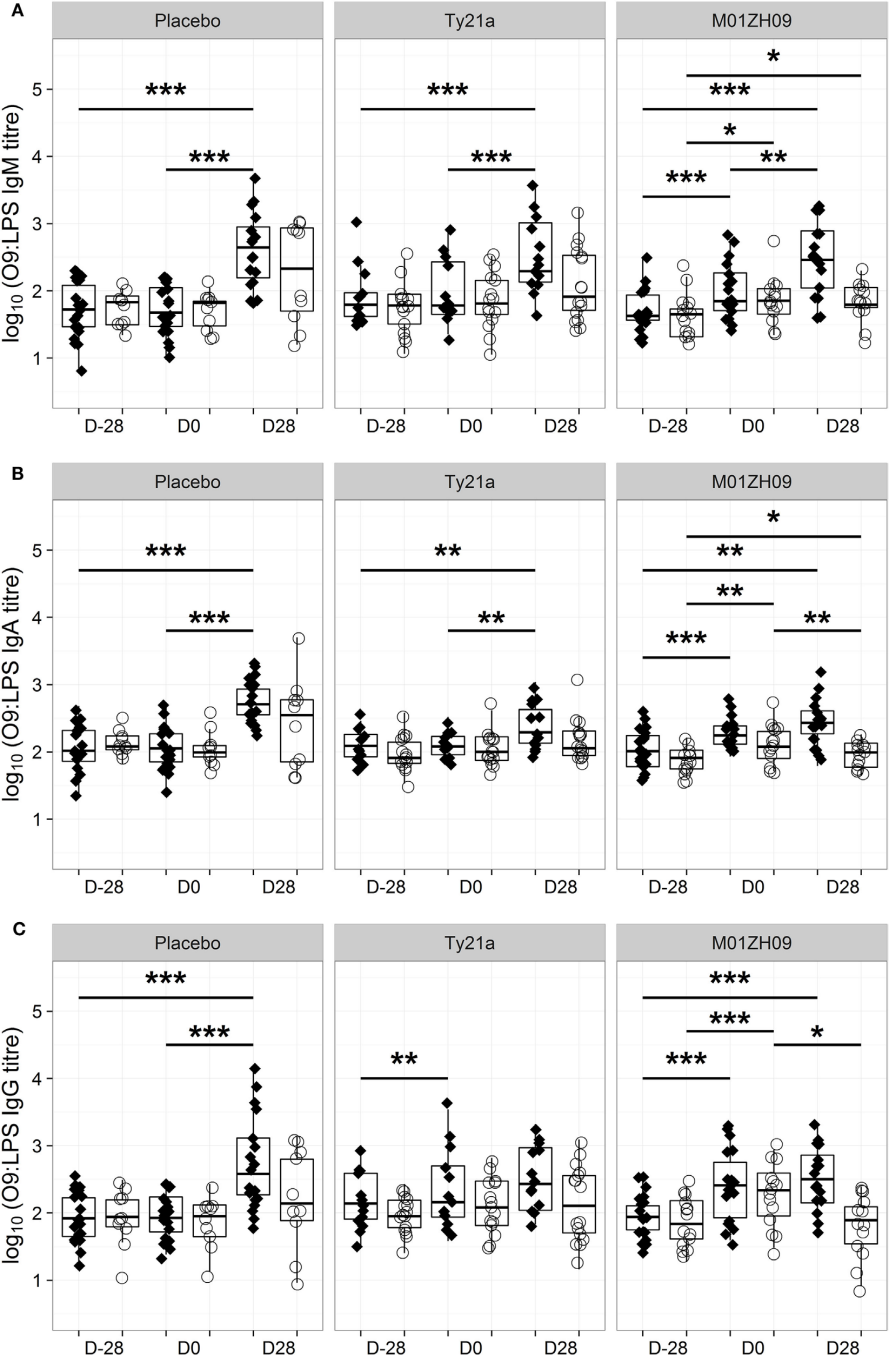
Kinetics of O9:LPS antibody induction. Anti-O9:LPS IgM **(A)**, IgA **(B)**, and IgG **(C)** antibodies at baseline (D28), on the day of challenge (D0) and at D28, in those with typhoid diagnosis (TD, diamonds), and no typhoid diagnosis (nTD, empty circles) within each study arm. *p < 0.05, **p < 0.01, ***p < 0.001. Significance was determined by Wilcoxon signed rank tests for paired value comparisons.

Consistent with these bactericidal activity data, the numbers of IgM, IgA, or IgG antibody-secreting cells specific to O9:LPS present in circulation 7 days after vaccination were not associated with protection from typhoid fever (Figures S1B–D in Supplementary Material). Similarly, the D0 IgM, IgA, or IgG antibody titers against O9:LPS or flagellin were not associated with protection against development of disease (Figures S1E–G and S2B–D in Supplementary Material). Anti-O9:LPS antibody titers showed a similar longitudinal profile to bactericidal activity (Figure 4). A notable exception was that anti-O9:LPS IgG significantly increased in Ty21a TD participants following vaccination (*p* = 0.009 at D0) but not in response to challenge.

## Discussion

This is the first direct investigation of the association between bactericidal activity and outcome of *S*. Typhi infection in humans, despite multiple *in vivo* studies of murine challenge with *Salmonella* species (10, 29, 30) and typhoid vaccine clinical trials (6, 7). Alongside recent investigations of *Shigella* spp. and pseudomonas infection (31, 32) this study points to a moderating role for bactericidal antibodies common to multiple bacterial diseases.

We reported previously that vaccination with Ty21a or with M01ZH09-induced IgG antibodies against O9:LPS (14). Further analyses here show that in the Ty21a group this rise in anti-LPS IgG is limited to TD participants. Earlier studies found that vaccination with Ty21a induces anti-LPS IgG antibodies in serum (9) that opsonize ahead of phagocytic killing (12), but their role in complement-mediated bactericidal killing had not been investigated. It is noteworthy that levels of serum bactericidal activity did not significantly change after Ty21a vaccination, even in TD participants, perhaps reflecting the contribution of additional uncharacterized mutations in Ty21a (17). M01ZH09 induced significant rises in anti-O9:LPS IgG, IgA, and IgM titers and in bactericidal activity, consistent with previous immunogenicity studies (16, 33). We observed that these rises were highest in the group with TD after challenge.

Bactericidal activity is an established correlate of protection for *Neisseria meningitidis* and used in vaccine licensure (34, 35). Bactericidal antibodies have been associated with age-related decrease in incidence of invasive non-typhoidal *Salmonella* (NTS) disease in African children and an age-dependent increase in bactericidal activity titer has also been documented in areas of high typhoid incidence (36–38). However, in our study the depletion of antibodies against O9:LPS abrogated bactericidal activity so it does not seem likely that either the presence, or bactericidal activity, of anti-LPS antibodies confers protection against invasion by *S*. Typhi. The data presented here do not support the assumption that these functional antibodies have an important role in sterile immunity against *S*. Typhi in adults.

Consistent with an inability to prevent disease oral challenge with wild-type *S*. Typhi in naïve volunteers induced anti-LPS IgG, IgA, and IgM and bactericidal antibodies but the responses were limited to those who developed typhoid infection, as shown in previous studies of anti-LPS antibodies (15, 21). Similarly, Ty21a vaccine recipients who developed typhoid generated anti-LPS IgG and bactericidal antibodies in response to oral challenge. In contrast, in both Ty21a vaccinated and naïve individuals the exposure to LPS during invasive infection did not induce a bactericidal response among those who are challenged, and presumably have at least mucosal exposure to the pathogen, but do not succumb to infection.

The participants who received M01ZH09 vaccine also developed a rise in anti-LPS IgM antibodies following infection but no further rise in anti-LPS IgG, IgA, or bactericidal activity was observed. Repeat vaccination in a relatively short period of time can induce hyporesponsiveness to polysaccharide antigens (39, 40) and anergy to protein superantigens (41), both associated with saturation of the immune response and presumably a negative feedback control to avoid overstimulation of B cells. However, bactericidal antibody titers in the M01ZH09 group were lower than those eventually attained in naive volunteers and Ty21a vaccine recipients. It seems more likely that the reduced bacterial load due to increased bactericidal activity from vaccination resulted in reduced exposure to LPS in this group, thus blunting any further response. This is supported by significantly higher bactericidal titers in the M01ZH09 group than in the Ty21a volunteers at D0.

Variants of O9:LPS antigen are abundant in the Enterobacteriaceae, including gut commensals, which may explain the presence of baseline bactericidal activity and the strong boost upon challenge in the placebo and even the Ty21a subgroups. Consistent with our data, others have shown bactericidal activity resulting from anti-LPS antibodies in sera from mice immunized with *S*. Paratyphi A Δ*guaBA* Δ*clpX* or *S*. Typhi Δ*guaBA* Δ*htrA* strains or invasive NTS isolates (10, 11, 29, 33). The serum bactericidal activity after vaccination or infection with *S*. Typhi is likely associated with anti-O9:LPS IgM or IgG complement-binding antibodies (42), as also observed with anti-O:LPS-mediated bactericidal activity against NTS. Although secretory IgA activates the mannan-binding lectin pathway (43), depletion of IgA from our samples did not significantly affect bactericidal activity. In studies examining the role of bactericidal activity against NTS in African adults with or without HIV infection, bactericidal activity was not seen with anti-O:LPS IgA and the bactericidal properties of sera depended on the relative titers of anti-O:LPS isotypes so that high titers of IgA antibodies correlated with absence of bactericidal activity (37, 44). Other mechanisms may be at play for the absence of protection from typhoid challenge we observed.

Despite the absence of protection, among vaccinated participants the delay in onset of infection associated with higher bactericidal antibody titer suggests that these antibodies may have an important role in reducing the bacterial load during infection, either at the point of invasion or during multiplication after the primary bacteremia. Additionally, the observed association with a reduction in disease manifestations and plasma cytokines suggests a role for these antibodies in reducing inflammation and symptom severity. In previous CHIM studies of both typhoid and paratyphoid infection (15, 21), we have shown a relationship between challenge dose and duration of the incubation period as well as a challenge dose-dependent association with blood bacterial load at diagnosis and anti-O:LPS titers. It is thus possible that the bactericidal activity of antibodies produced by vaccination reduced the bacterial load in the initial stages of infection and this in turn may have delayed disease onset or reduced other disease symptoms. This is consistent with a very recent investigation of shigellosis in a human challenge model and studies on patients with chronic *Pseudomonas aeruginosa* respiratory infections (31, 32). In these studies reduced SBA activity was associated with increased disease severity using parameters such as stool symptomatology and temperature (32) or lung forced expiratory volume (31). Bactericidal activity may also result in lower bacterial burden in these cases.

Our analysis, which relies on gaining multiple samples from the same individuals, is limited by low statistical power. We were not able to determine the role of anti-Vi antibodies since the immune response to the capsular antigen was negligible. Nevertheless, the data imply that O9:LPS might not be the ideal antigen for new typhoid vaccines as neither the concentration of anti-O9:LPS antibody, frequency of anti-O9:LPS ASC nor bactericidal antibody titers at time of challenge correlated with protection in our model. Recent vaccine development for typhoid fever has focused on Vi-conjugate vaccines, but efforts to develop vaccines against invasive NTS strains (30, 45) and against *S*. Paratyphi have focused on O:LPS antigens (46). In contrast to *S*. Typhi, none of these pathogens are encapsulated with polysaccharide. For *S*. Paratyphi B there is evidence from field trials that Ty21a provides cross-protection (47) and in our CHIM there is also evidence that anti-O2:LPS IgG titers may correlate with protection against *S*. Paratyphi A (21), thus examination of O polysaccharide-conjugate vaccines currently in development (46) in a CHIM is warranted. No such model for invasive NTS is available, and would raise some ethical concerns (13), to evaluate the role of bactericidal activity mediated by anti-O:LPS antibodies observed *in vitro* and *in vivo* (29, 37, 44). Future studies examining other antibody properties and cell-mediated mechanisms would enhance understanding of immunity to invasive *Salmonellae*.

We sought to identify the role of bactericidal antibodies in serum following challenge with *S*. Typhi in a human challenge model. We identified that anti-LPS antibodies mediate a bactericidal effect that delays onset of infection and reduces disease severity and inflammation. This directs future vaccine studies toward other antigens or mechanisms of protection against typhoid.

## Ethics Statement

A randomized, double-blind, placebo-controlled trial was performed at the Centre for Clinical Vaccinology and Tropical Medicine, Churchill Hospital, Oxford, UK (clinicaltrials.gov NCT679172; EudraCT 2011-000381-35) (14). This study was carried out in accordance with the recommendations of the Oxford University Clinical Trials and Research Governance Department, and the protocol approved by NRES South Central––Oxford A (11/SC/0302) and conducted in accordance with the declaration of Helsinki (2008) and the International Conference of Harmonization of Good Clinical Practice guidelines. An independent Data Monitoring and Safety Committee monitored the trial. To perform the assays described, human complement was collected in a parallel, prospective observational study (clinicaltrials.gov NCT01945307; 13/SC/0375). All subjects gave written informed consent in accordance with the Declaration of Helsinki.

## Author Contributions

HT-B and HJ contributed to the drafting of the manuscript. CB, TD, and AP conceived and designed the work. HJ, HT-B, CJ, EJ, SS, RS, AE, PK, TV, and NT acquired data for the work. HJ, HT-B, and UG analyzed the work. HJ, HT-B, CJ, TD, SB, and AP interpreted data for the work. All authors were involved in article revision and approved the final published version. All authors agree to be accountable for all aspects of the work in ensuring that questions related to the accuracy or integrity of any part of the work are appropriately investigated and resolved.

## Conflict of Interest Statement

AP has previously undertaken clinical studies on behalf of the University of Oxford, which were funded by vaccine manufacturers, but no longer does so. His department has received unrestricted educational grants from vaccine manufacturers to support delivery of a course on childhood infection. AP is chair of the UK Department of Health’s (DH) Joint Committee on Vaccination and Immunization (JCVI), and a member of WHOs Strategic Advisory Group of Experts, but the views expressed in this manuscript do not necessarily represent the views of JCVI or DH or WHO. All other authors declare that the research was conducted in the absence of any commercial or financial relationships that could be construed as a potential conflict of interest.

## Supplementary Material

Figure S1 Bactericidal antibodies do not protect against typhoid infection. **(A)** SBA titer on the day of challenge, in those with typhoid diagnosis (TD) and no typhoid diagnosis (nTD) within each study arm. Significance was determined by Mann–Whitney tests. **(B–D)** Numbers of cells secreting IgM **(B)**, IgA **(C)**, or IgG **(D)** antibodies against O9:LPS 7 days after vaccination, in TD and nTD groups across all study arms. Significance was determined by Mann–Whitney tests. **(E–G)** Antibody titers against O9:LPS IgM **(E)**, O9:LPS IgA **(F)**, and O9:LPS IgG **(G)** on the day of challenge (D0), in TD and nTD groups across all study arms. Significance was determined by Mann–Whitney tests.

Figure S2 Anti-Vi and anti-flagellin antibodies do not protect against typhoid infection. Antibody titers against Vi IgG **(A)**, flagellin (H) IgM **(B)**, H IgA **(C)**, and H IgG **(D)** on the day of challenge (D0), in TD and nTD groups across all study arms. Significance was determined by Mann–Whitney tests.
