# Supplementary material for: Salmonella Typhi Bactericidal Antibodies Reduce Disease Severity but Do Not Protect against Typhoid Fever in a Controlled Human Infection Model
Source: Front Immunol. 2018 Jan 17;8:1916. doi: 10.3389/fimmu.2017.01916 (PMC5776093; doi:10.3389/fimmu.2017.01916)
Supplement: Supplementary file 1 [file Table_1.docx]

Supplementary Material

*Salmonella* Typhi bactericidal antibodies reduce infection severity but do not protect against typhoid fever

**Helene B Juel^1,2a^, Helena B Thomaides-Brears^1a*^, Thomas C Darton^1,3^, Claire Jones^1^, Elizabeth Jones^1^, Sonu Shrestha^1^, Rebecca Sie^1^, Andrew Eustace^1^, Ushma Galal^4^, Prathiba Kurupati^5^, Tan T Van^6^, Nga TV Thieu^6^, Stephen Baker ^6,7,8^, Christoph J Blohmke^1^, Andrew J Pollard^1^**

^1^Oxford Vaccine Group, Department of Paediatrics, University of Oxford and the NIHR Oxford Biomedical Research Centre, Oxford, United Kingdom

^2^Statens Serum Institut, Copenhagen, Denmark

^3^Department of Infection, Immunity and Cardiovascular Disease, University of Sheffield, Sheffield, United Kingdom

^4^Nuffield Department of Primary Care Health Sciences, Clinical Trials Unit, University of Oxford, Oxford, United Kingdom

^5^Weatherall Institute of Molecular Medicine, University of Oxford, Oxford, United Kingdom

^6^The Hospital for Tropical Diseases, Wellcome Trust Major Overseas Programme, Oxford University Clinical Research Unit, Ho Chi Minh City, Vietnam

^7^Centre for Tropical Medicine and Global Health, Nuffield Department of Medicine, University of Oxford, Oxford, United Kingdom

^8^The Department of Medicine, University of Cambridge, Cambridge, United Kingdom

^a^These first authors contributed equally to the study.

*** Correspondence:**Helena B. Thomaides-Brears
helena.thomaides-brears@paediatrics.ox.ac.uk

Table S1:

Antibody titres in human complement source used in the SBA, pre- and post-incubation with *S.* Typhi Quailes.

| **Antibody** | **Titre pre-incubation**  **(EU/ml)** | **Titre post-incubation**  **(EU/ml)** | **% Depletion** |
| --- | --- | --- | --- |
| O9:LPS IgG | 1828.3 | 580.8 | 68.23 |
| O9:LPS IgA | 4016.7 | 859.2 | 78.61 |
| Vi IgG | 47.16 | 2.98 | 93.68 |
