# Supplementary material for: Salmonella Typhi Bactericidal Antibodies Reduce Disease Severity but Do Not Protect against Typhoid Fever in a Controlled Human Infection Model
Source: Front Immunol. 2018 Jan 17;8:1916. doi: 10.3389/fimmu.2017.01916 (PMC5776093; doi:10.3389/fimmu.2017.01916)
Supplement: Supplementary file 2 [file Table_2.docx]

Supplementary Material

*Salmonella* Typhi bactericidal antibodies reduce infection severity but do not protect against typhoid fever

**Helene B Juel^1,2a^, Helena B Thomaides-Brears^1a*^, Thomas C Darton^1,3^, Claire Jones^1^, Elizabeth Jones^1^, Sonu Shrestha^1^, Rebecca Sie^1^, Andrew Eustace^1^, Ushma Galal^4^, Prathiba Kurupati^5^, Tan T Van^6^, Nga TV Thieu^6^, Stephen Baker ^6,7,8^, Christoph J Blohmke^1^, Andrew J Pollard^1^**

^1^Oxford Vaccine Group, Department of Paediatrics, University of Oxford and the NIHR Oxford Biomedical Research Centre, Oxford, United Kingdom

^2^Statens Serum Institut, Copenhagen, Denmark

^3^Department of Infection, Immunity and Cardiovascular Disease, University of Sheffield, Sheffield, United Kingdom

^4^Nuffield Department of Primary Care Health Sciences, Clinical Trials Unit, University of Oxford, Oxford, United Kingdom

^5^Weatherall Institute of Molecular Medicine, University of Oxford, Oxford, United Kingdom

^6^The Hospital for Tropical Diseases, Wellcome Trust Major Overseas Programme, Oxford University Clinical Research Unit, Ho Chi Minh City, Vietnam

^7^Centre for Tropical Medicine and Global Health, Nuffield Department of Medicine, University of Oxford, Oxford, United Kingdom

^8^The Department of Medicine, University of Cambridge, Cambridge, United Kingdom

^a^These first authors contributed equally to the study.

*** Correspondence:**Helena B. Thomaides-Brears
helena.thomaides-brears@paediatrics.ox.ac.uk

Table S2:

Summary statistics of log_10_SBA titres across all study arms at pre-vaccination baseline D-28.

| **Study arm** | **Median** | **IQR** | **Min** | **Max** |
| --- | --- | --- | --- | --- |
| Placebo | 3.48 | 2.91-4.08 | 2.40 | 4.81 |
| Ty21a | 3.48 | 3.00-3.78 | 2.27 | 4.38 |
| M01ZH09 | 3.39 | 3.00-3.60 | 2.10 | 4.08 |
