# Supplementary figures and images for: Salmonella Typhi Bactericidal Antibodies Reduce Disease Severity but Do Not Protect against Typhoid Fever in a Controlled Human Infection Model
Source: Front Immunol. 2018 Jan 17;8:1916. doi: 10.3389/fimmu.2017.01916 (PMC5776093; doi:10.3389/fimmu.2017.01916)

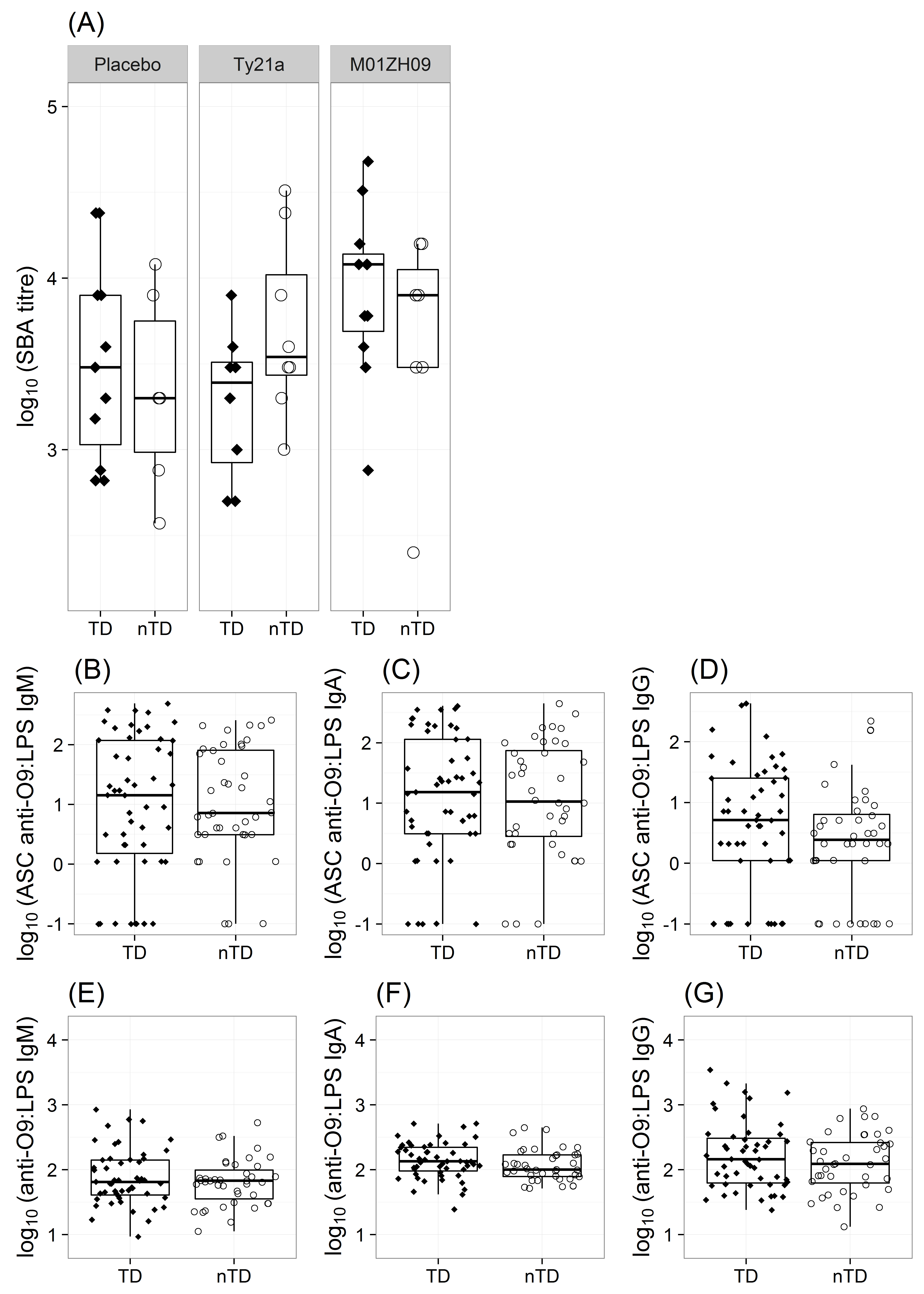

Supplement: Figure S1 — Bactericidal antibodies do not protect against typhoid infection. (A) SBA titer on the day of challenge, in those with typhoid diagnosis (TD) and no typhoid diagnosis (nTD) within each study arm. Significance was determined by Mann–Whitney tests. (B–D) Numbers of cells secreting IgM (B), IgA (C), or IgG (D) antibodies against O9:LPS 7 days after vaccination, in TD and nTD groups across all study arms. Significance was determined by Mann–Whitney tests. (E–G) Antibody titers against O9:LPS IgM (E), O9:LPS IgA (F), and O9:LPS IgG (G) on the day of challenge (D0), in TD and nTD groups across all study arms. Significance was determined by Mann–Whitney tests. [file Image_1.tiff]

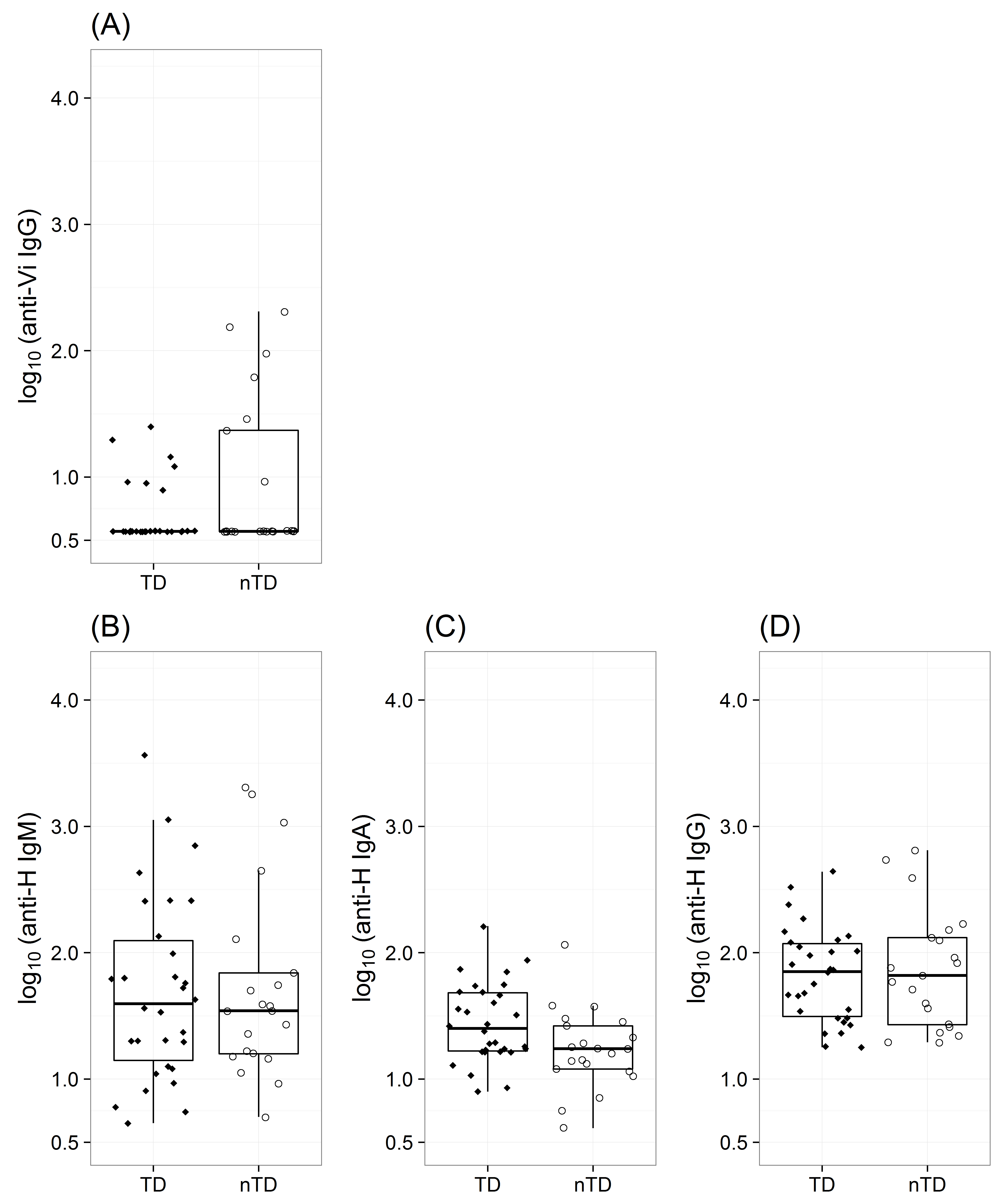

Supplement: Figure S2 — Anti-Vi and anti-flagellin antibodies do not protect against typhoid infection. Antibody titers against Vi IgG (A), flagellin (H) IgM (B), H IgA (C), and H IgG (D) on the day of challenge (D0), in TD and nTD groups across all study arms. Significance was determined by Mann–Whitney tests. [file Image_2.tiff]
